# Heterologous Expression of Cyanobacterial Cyanase Gene (*CYN*) in Microalga *Chlamydomonas reinhardtii* for Bioremediation of Cyanide Pollution

**DOI:** 10.3390/biology11101420

**Published:** 2022-09-29

**Authors:** Shaimaa S. Sobieh, Rasha Abed El-Gammal, Wafaa S. Abu El-Kheir, Alia A. El-Sheimy, Alaa A. Said, Yassein M. El-Ayouty

**Affiliations:** 1 Botany Department, Faculty of Women for Arts, Science and Education, Ain Shams University, Cairo 11511, Egypt; 2 Botany and Microbiology Department, Faculty of Science, Zagazig University, Sharkia 44671, Egypt

**Keywords:** cyanide pollution, genetic transformation technique, *Agrobacterium tumefaciens*, transgenic *Chlamydomonas reinhardtii*, cyanase

## Abstract

**Simple Summary:**

Cyanide is a known toxic compound produced through natural and anthropogenic activities. Water can be polluted by cyanide ions through wastewater effluents. In high concentrations, cyanide is considered a strong metabolic inhibitor and can cause inhibition in mitochondrial complex IV (cytochrome c oxidase), and its assimilation can result in chronic poisoning and/or acute poisoning to humans and animals. Bioremediation systems involving the usage of transgenic algal systems have become preferable alternatives for the detoxification of cyanide contamination due to the accumulation and the biosorbent efficiency of transgenic *Chlamydomonas* in the removal of KCNO from fresh water.

**Abstract:**

Recombinant DNA technology offered the creation of new combinations of DNA segments that are not found together in nature. The present study aimed to produce an ecofriendly bioremediation model to remediate cyanide pollution from a polluted marine system. Cyanide is a known toxic compound produced through natural and anthropogenic activities. An *Agrobacterium-tumefaciens*-mediated genetic transformation technique was used to generate transformed *Chlamydomonas reinhardtii* using plant expression vector pTRA-K-cTp carries isolated coding sequence of the cyanobacterial cyanase gene (*CYN*) isolated from *Synechococcus elongatus* (PCC6803). qRT-PCR analysis showed the overexpression of *CYN* in transgenic *C. reinhardtii*, as compared with the respective wild type. Growth parameters and biochemical analyses were performed under cyanide stress conditions using transgenic and wild *C. reinhardtii* for evaluating the effect of the presence of the cyanobacterial cyanase gene in algae. The transgenic *C. reinhardtii* strain (*TC. reinhardtii-2*) showed promising results for cyanide bioremediation in polluted water samples. Cyanide depletion assays and algal growth showed a significant resistance in the transgenic type against cyanide stress, as compared to the wild type. Genetically modified alga showed the ability to phytoremediate a high level of potassium cyanide (up to150 mg/L), as compared to the wild type. The presence of the *CYN* gene has induced a protection response in *TC. Reinhardtii-2*, which was shown in the results of growth parameter analyses. Therefore, the present study affirms that transgenic *C. reinhardtii by* the *CYN* coding gene is a potential effective ecofriendly bioremediator model for the remediation of cyanide pollutants in fresh water.

## 1. Introduction

The world is facing several problems by varieties of pollutants and contaminates from various developmental activities. The earth’s atmosphere and natural waters are polluted by municipal, industrial, and agricultural wastes [1]. Nowadays in Egypt, water pollution by cyanides is a potent threat on humans, plants, and animals [2]. Cyanide (CN) is a chemical group containing one carbon atom triply bonded to a nitrogen atom, and it can form other chemical complexes with several atoms [3]. In the environment, cyanide can occur both naturally or because of anthropogenic activities, and many of its complexes are toxic and rapid-acting substances [4]. Aquatic environments can be polluted by cyanide ions through wastewater effluents [3]. Cyanide occurs in aquatic environments as cyanide ion, hydrogen cyanide, simple cyanides, metallo-cyanide complexes, and as simple chain and complex ring compounds [3]. In high concentrations, cyanide is considered a strong metabolic inhibitor and can cause inhibition in mitochondrial complex IV (cytochrome c oxidase), and its assimilation can result in chronic poisoning and/or acute poisoning to humans and animals [3]. Moreover, fish and other aquatic life are killed by cyanide concentrations in the microgram per liter range [5]. Every year, the world consumes around 360 thousand ton of sodium cyanide (NaCN), 33% of which is used in the recovery of gold and silver, whereas the production of HCN is about 1.4 million tones yearly [6].

In consequence of high cyanide toxicity, the US Environmental Protection Agency (USEPA) has set the permissible limit of cyanide (200 ppb) for drinking water and 500 ppb for aquatic-biota water [7], whereas the World Health Organization (WHO) has proposed a maximum concentration limit (70 ppb) of cyanide in drinking water [8]. Therefore, cyanide-containing effluents must be treated or remediated to reduce its level in contaminated soil and water to the permissible limit [9]. Cyanide compounds are detoxified mainly by chemical treatments involving chlorination reactions [10]. However, these chemical treatments produce hazardous byproducts, so they have to be replaced by other ecofriendly methods [11].

Bioremediation systems involving the usage of plants or microorganisms are ecofriendly and affordable alternatives [10]. Gurbuz et al. [12] showed that bioremediation using algal systems became preferable alternatives for the detoxification of cyanide contamination because of their volume ratios, efficient uptake, and storage systems.

In living organisms, cyanase enzyme is important for the degradation and/or removal of the toxic cyanide and cyanate compounds affecting their growth [13]. Cyanase (EC 4.2.1.104) is bacterial enzyme that can transform cyanide into non-toxic compounds and use them as carbon and nitrogen sources [14,15]. Interestingly, some bacteria were shown to grow on cyanate as sole nitrogen and carbon sources due to the endogenous cyanase activity [16]. Jin et al. [17] reported the direct relationship between the detoxification of CN by algal cells and the concentration of CN due to the accumulation of KCN by algal cells until saturation in a particular concentration. Moreover, the biosorbent efficiency of transgenic *Chlamydomonas* in the removal of KCNO from an aqueous solution was previously reported [18].

Generally, microorganisms are genetically engineered by inserting foreign genes into their genome. Several methods for algal transformation have been developed. Among these methods, *Agrobacterium-tumefaciens*-mediated transformation is known as a highly efficient and stable method [19]. Several reports have been made on the successful transformation of the green algae, *Chlamydomonas, Volvox, Chlorella, Dunaliella*, *and Haematococcus*. Among the *Chlamydomonas* species, *C. reinhardtii* has attracted more attention as a model for studying biological systems because it is the most biologically characterized *Chlamydomonas* [20]. Research into recombinant protein production, such as the expression of enzymes, proteins, human growth factors, antibodies, and vaccines, in *Chlamydomonas reinhardtii* has increased recently [20].

The present study aimed to bioremediate cyanide pollution in fresh water using transgenic *Chlamydomonas reinhardtii* by transferring the cyanobacterial *cyanase* gene (CYN, gi16329170) into the nuclear genome of *Chlamydomonas reinhardtii* via the *Agrobacterium-tumefaciens*-mediated transformation method. The growth parameters of transgenic *C. reinhardtii* and wild lines under cyanate stress have been analyzed, and the expression level of the cyanase gene in wild and transgenic lines was estimated using a quantitative real-time polymerase chain reaction (PCR).

## 2. Materials and Methods

### 2.1. Cultivation of Chlamydomonas Reinhardtii

*Chlamydomonas reinhardtii* strain, CC-124 (mt−), was cultivated according to ELgammal et al. [21]. Solid tris acetate phosphate (TAP) medium was supplemented with 1.5 percent (*w*/*v*) agar and (50 μg/mL) kanamycin (required for selection of transgenic strain, which can only survive in the presence of kanamycin). TAP medium was inoculated by 5 mL of *C. reinhardtii*, and then incubated in a growth chamber under a photoperiod of light: dark cycles (16–8) at a temperature of 25 ± 2 °C to select transformed *C. reinhardtii* colonies. The optimum light intensity, 6.75 µmol photons m^−2^ s^−1^, was supplied with cool white fluorescent tubes for 26 days. The grown colonies in presence of kanamycin were harvested, and then inoculated in sterilized fresh TAP medium and grown under the former growth conditions.

### 2.2. Transformation of E. coli (DH5α) by Synechococcus Elongatus Cyanase Gene

**Figure 1:**
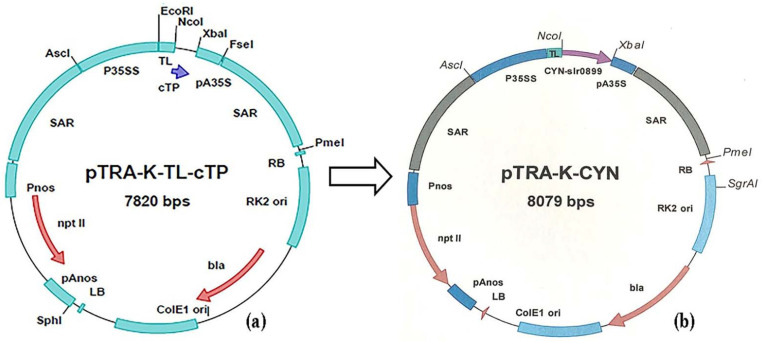
(**a**) Structure of pTRA-K-TL-cTP binary expression vector. (**b**) Structure of pTRA-K-CYN plasmid DNA. *Synechococcus elongatus* PCC 6803 cyanase gene was cloned into the binary plant expression vector, pTRA-K (gi13508478), between NcoI and XbaI sites. *CYN* gene cassette is flanked by scaffold attachment regions (SAR) of the tobacco RB7 gene tobacco leader peptide (TL), and 3′ UTR of CaMV 35S (pA35S). LB/RB: left and right border sequences of nopaline-Ti-plasmids pTiT37. pAnos: polyadenylation signal of nopaline synthetase gene from *A. tumefaciens*. NptII: neomycin phosphotransferase type II refers to resistance to kanamycin. pnos: promoter of nopaline synthase gene from *A. tumefaciens*.

The genomic DNA of cyanobacterial algal *Synechococcus elongatus* (PCC 6803) was isolated according to Varela-Álvarez et al. [22]. It was used as a template for the PCR amplification of the *Cyanase* gene coding sequence (CYN, gi16329170, E.C.4.2.1.104) using *CYN*-gene-specific primers (Metabion): CYN1-NcoI-Fw (5′-ATGGCCATGGCTGGCACTGAAATTTC-3′) and CYN1-Xba1-Rev (5′-GTCACTCGAGCCATTTCT TGTAGGGTAA-3′). A 25-μL reaction mixture, including 0.5 μL dNTPs (100 μM), 0.25 μL Taq DNA polymerase (5 U μL^–^^1^), 0.5 μL of each primer (10 pmol), 0.5 μL of MgCl_2_ (25 mM), and 2.5 μL of 10X PCR reaction buffer, was mixed with 3 μL template DNA (50 ng μL^–1^) and 17.5 μL bi-distilled H_2_O. PCR amplification was performed with a Techni TC-312 PCR (Stafford, UK system). PCR was programmed as follows: 1 cycle at 94 °C for 5 min (initial denaturation), followed by 35 cycles for 30 s at 94 °C (denaturation), 30 s at 60 °C (annealing), 1 min at 72 °C (extension), followed by one final extension of 5 min at 72 °C, and cooling to a temperature of 4 °C (infinite). PCR products were visualized on a 1% (*w*/*v*) agarose gel containing ethidium bromide (0.5 μg/mL). The amplified PCR products of the *CYN* gene were purified using a MEGA quick-spin PCR & Agarose Gel DNA Extraction kit (iNtRON; Korea). The purified *CYN* fragment was ligated into the pTRA-K-cTP vector. The vector, pTRA-K-cTP (Figure 1a), was subjected to restriction digestion using *NcoI* and *XbaI* restriction enzymes for 2 h at 37 °C. The restricted products were then column-purified to remove the resulting small end fragments after enzymatic digestion. Amplified *CYN* genes were cloned into the vector, pTRA-K-cTP, between NcoI and XbaI sites (Figure 1b). The *CYN* gene cassette is flanked by scaffold attachment regions (SAR) of the tobacco RB7 gene tobacco leader peptide (TL); *CYN* gene expression is controlled by CaM promoter (p35SS), 5′UTR of the Cab22L tobacco leader peptide (TL), and 3′UTR of CaMV 35S (pA35S) [23]. The neomycin phosphotransferase type II (*NptII*) gene was used to select transgenic *C. reinhardtii* [24]. Ligation was performed using T4-DNA ligase at 16 °C overnight. The ligation mixtures were used directly for the transformation of *E. coli* (DH5α) bacterial cells according to Liu and Rashidbaigi [25]. The *E. coli* strain, DH5α, was used for the establishment and cloning of the plasmid vector (pTRA-K-CYN). Then, the plasmid DNA was isolated from positive transformed DH5α colonies, and restricted with *NcoI* and *XbaI* restriction enzymes.

### 2.3. Transformation of Agrobacterium Tumefaciens by pTRAK-CYN vector

*Agrobacterium tumefaciens* (GV3101) was cultured in yeast extract medium (YEB medium) containing 100 µg/mL rifampicin (Rif) and 25 µg/mL kanamycin (Kan) (YEB-Rif-Kan) on a shaker for 2 days at 28 °C until OD600 reached 1–1.5. The cells were spun down by centrifugation at 4000× *g* at 4 °C for 5 min. The culture medium was decanted, and the cells were washed three times with 100 mL, 50 mL, and 25 mL of 100 mM CaCl_2_. pTRAK-CYN vector (0.2–1.0 µg) in dist. H_2_O was added to the aliquot of *Agrobacterium* electro-competent cells and incubated on ice for 30 min. The cell/DNA mixture was incubated at 37 °C for 10 min and placed directly on ice for 2 min. A volume of 1 mL of YEB medium was added immediately to the tubes containing the heat-shocked bacteria [19]. The transformed cells were incubated at 28 °C for 2 h with continuous shaking. Transformed *A. tumefaciens*, with the pTRAK-CYN plasmid, was plated onto YEB-agar plates supplemented with rifampicin, kanamycin, and ampicillin, and then incubated at 28 °C for a further 2–3 days. Transformed *A. tumefaciens*, with the pTRAK-CYN plasmid, was cultured in liquid YEB medium supplemented with 25 µg/L kanamycin and 100 µg/L rifampicin at 28 °C. Then, the transformed *A. tumefaciens* cells were spun down at 4000× *g* for 10 min at 4 °C and resuspended in 250 μL liquid TAP medium until OD600 equaled 0.6 to produce virulent *A. tumefaciens.*

### 2.4. Formation and Selection of Transgenic Chlamydomonas reinhardtii

Wild-type *C. reinhardtii* was transformed by the pTRAK-CYN vector via the *Agrobacterium tumefaciens* according to Kumar and Rajam [26]. A colony of wild-type *C. reinhardtii* was inoculated into liquid TAP medium and allowed to grow to the log phase. Cells were then plated on solid TAP medium and incubated under continuous light for 2 days until forming a lawn of cells. For the transformation of *C. reinhardtii,* the transformed *A. tumefaciens* suspension was co-cultivated with *C. reinhardtii* cells and grown on the agar plates for 2 h at 28 °C, and then kept overnight in a dark chamber at 25 °C. All cells of *C. reinhardtii* were washed twice with liquid TAP medium supplemented with 500 mg/L cefotaxime to eliminate *Agrobacterium*. The presence of the *CYN* transgene in the transformed *C. reinhardtii* colonies was checked by a colony PCR test using *CYN*-specific primers.

### 2.5. Quantitative Real-Time PCR Analysis

Total RNA was extracted from wild and transgenic *C. reinhardtii* in accordance with Chomczynski and Mackey [27]. Isolated RNA samples were digested with DNase enzyme to remove any DNA contamination. One unit of DNase I (Roche Applied Science) per microgram of RNA and MgCl_2_ to a final concentration of 2 mM were added [26]. The purity of the RNA samples was visualized on 2% (*w*/*v*) agarose gel containing ethidium bromide. The first-strand cDNA was synthesized from the isolated RNA from the transgenic algae, as reported by Niessen et al. [28]. About 400 ng of the RNA sample were mixed with 10 µL bi-distilled H_2_O and 1µL Oligo(dT) primer (10 pmol/µL). The reaction mixture was incubated 5 min at 65 °C and cooled down on ice; 2 µL dNTPs, 1.5 units of reverse transcriptase enzyme Moloney murine leukemia virus reverse transcriptase (MMLV-RT) (Sigma-Aldrich), and 4 µL MMLV-RT buffer (5× reverse transcriptase buffer) were added, and incubated for 40 min at 37 °C. Finally, the enzyme activity was stopped by heating at 70 °C for 15 min. Quantitative real-time analysis of the *CYN* gene was performed using a qPCR^TM^ Core Kit for SYBR^®^ Green I (Eurogentec) in a Step One™ Real Time PCR System (Applied Biosystems, Waltham, MA, USA) following the manufacturer’s instructions, in the presence of SYBR Green as the fluorescent dye. The specific oligonucleotides for the *CYN* gene (Metabion) were CYN RT-PCR Fw (5′-GGG AAT CAC GTT TGCT GAT TT-3′) and CYN RT-PCR Rev (5′-AAG TTT CTC CGC CTC ATC AA-3′), with a final primer concentration of 10 nM in the reaction mixture. As internal control, *Actin2* was used using the specific primers: Actin2 Fw (5′- GCG ATG TGG ACA TCC GCA AG-3′) and Actin2 Rev (5′-GGG CCG TGA TCT CCT TGC TC-3′), with a final primer concentration of 10 nM in the reaction mixture. The MgCl_2_ concentration was 2.25 mM and the dNTPs concentration was 200 μM. The amplification conditions for both *CYN* and *Actin2* were 1 cycle at 94 °C for 10 min (initial denaturation), followed by 40 cycles for 15 s at 94 °C (denaturation), 15 s at 55 °C (annealing), 1 min at 72 °C (extension), followed by one final extension of 5 min at 72 °C, and cooling to a temperature of 16 °C (infinite). The average expression was recorded from three independent replicates for each gene.

### 2.6. Comparison between Growth Parameters of Wild and Transgenic C. reinhardtii under Potassium Cyanide Stress

Transgenic *C. reinhardtii* for the cyanase gene in addition to the wild type were used for assessing several growth parameters under potassium cyanide stress. Equal volumes of wild and transgenic *C. reinhardtii* cells were added in 50 mL liquid TAP media supplemented with 25, 50, 100, 150, and 200 mg/L potassium cyanide (3 flasks for each concentration), and a concentration of 0 was used as a control under aseptic conditions. All samples were allowed to cultivate with continuous shaking for 26 days. The cell density for each line was recorded at 665 nm at regular interval periods of 48 h. Several growth parameters were calculated.

### 2.7. Mean Growth Rate (R) (Number of Divisions/Day)

The mean growth rate was calculated according to formula proposed by Robert [29].

R = [3.322/t_2_ − t_1_][Log N_2_ − Log N_1_], where: 3.322: growth constant, t_1_: zero time of experiment, t_2:_ the end time of experiment, N_1_:OD of cells/mL culture at t_1,_ N_2_: OD of cells/mL cultured at t_2_.

### 2.8. Relative Growth Rate (K\) Was Calculated according to Formula of Fogg [30]

K^\^ = Log N − Log N_o_/t, where: N is OD of cells/mL culture after time t (days), N_o_ is OD of cells/mL culture at initial time.

**Generation time (G)** is the time needed for doubling the number of cells. It was calculated according to the formula proposed by Fogg [30]. G = 0.301/K^\^, where: K^\^ is the relative growth rate.

### 2.9. Bioremediation of Potassium Cyanide by Wild and Transgenic C. reinhardtii

To prove the efficiency of transgenic *C. reinhardtii* to degrade and bioremediate potassium cyanide compared to the wild type, the degradation frequency by one transgenic *C. reinhardtii* strain and wild types in the presence of different concentrations of potassium cyanide was quantified. Six groups of liquid TAP medium were used in this assay. Each concentration of 25, 50, 100, 150, and 200 mg/l KCN were supplemented to three flasks containing 50 mL liquid TAP medium and three flasks for the control group, which had no KCN. About 5 mL (1 mL = 0.6 OD) from each algal type were inoculated into the 1 mL liquid TAP medium and incubated for 72 h under a light intensity of 80 μmol m^−2^ s^−1^ at 25 °C. A volume of 5 mL was taken from each group at different times (0, 6, 12, 24, 36, 48, and 72 h) for the determination of KCN residue by using picric acid [31]. The absorbance was measured at 520 nm against a reagent blank by a YQ00302 spectrophotometer.

The amount of KCN removal (mg/L) = C_o_ − C_r_

Where: C_o_ is the initial concentration of cyanide (mg/L), C_r_ is the residual concentration of cyanide (mg/L).

### 2.10. Statistical Analysis

The results were expressed as the mean of three replicates ± standard error (SE). The obtained data were analyzed statistically using a *t*-test to determine significant differences among the data. All statistical analyses were carried out using Microsoft Excel software (Microsoft Corporation, Washington, DC, USA).

## 3. Results

### 3.1. Transformation of E. coli (DH5α) by Synechococcus Elongatus Cyanase Gene

**Figure 2:**
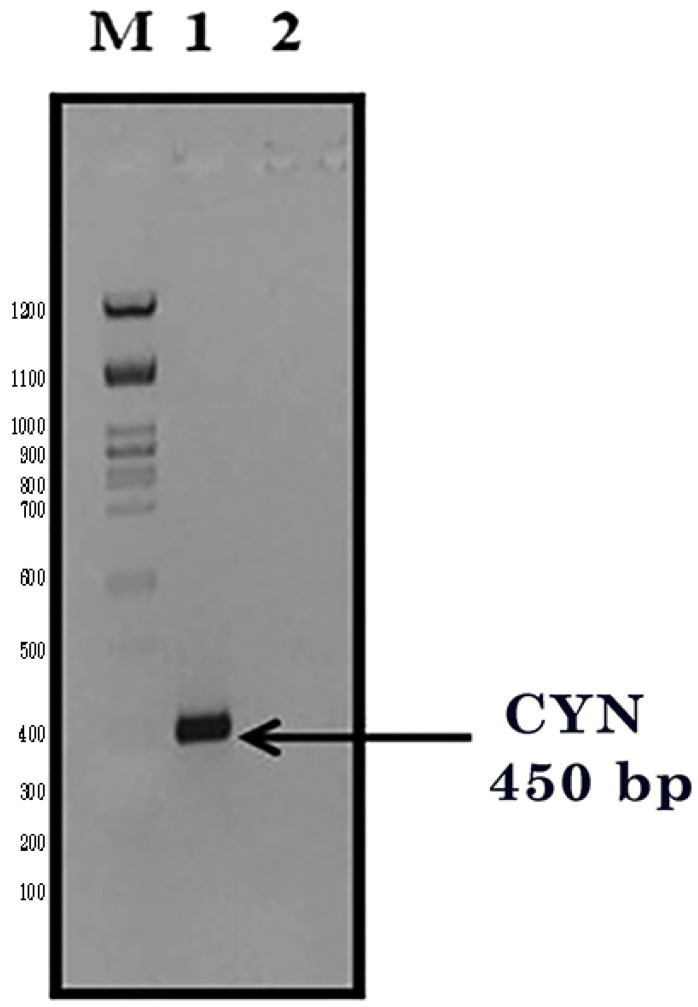
PCR product of the *CYN* amplification (450 bp). M: 100 bps DNA ladder, Lane 1: amplified *CYN* gene fragment, Lane 2: negative PCR control.

The cyanase gene (*CYN*) was isolated from the cyanobacterial *Synechococcus elongatus* (PCC6803). The *CYN* gene was composed of an open reading frame (ORF) of 450 base pairs that encoded a protein of 150 amino acids with a calculated molecular weight of 15 KDa. The *CYN* gene was amplified by PCR, and the PCR products were visualized on a 1% (*w*/*v*) agarose gel containing ethidium bromide. The PCR products gave the right expected product (450 bps) for *CYN* against 100 bps of the DNA marker (Figure 2). Then, purified *CYN* fragments were ligated into the plant expression vector, pTRA-K-cTP. To produce transgenic *E. coli* (DH5α) expressing the external *CYN* gene, the ligation mixtures were transformed into freshly prepared CaCl_2_ chemically competent DH5α bacteria.

**Figure 3:**
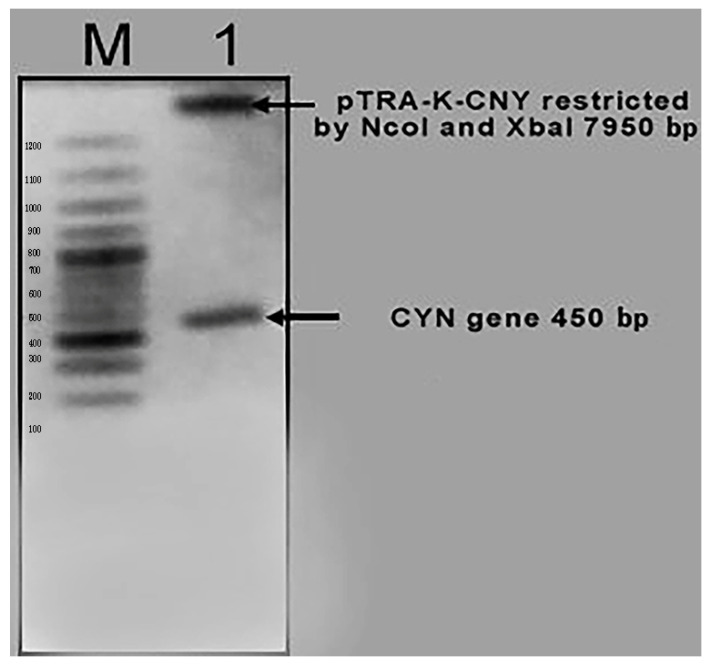
The generation of the right pTRA- K-CYN clones with right pattern, as expected (450 bp + 7950 bp).

Plasmid DNA was isolated from the overnight culture of transformed DH5α, and was then restricted with *NcoI* and *XbaI* restriction enzymes. Aliquots of the restricted samples were visualized in a 1% (*w*/*v*) agarose gel containing ethidium bromide. The obtained result of the isolated plasmid indicates the generation of the right pTRA- K-CYN clones (450 bp + 7950 bp) (Figure 3).

The sequencing of *CYN* fragments that were cloned into the pTRA-K-cTP plant expression vector was performed using vector general primers named pS5′-FW and pS3′-Rev. The sequencing results were compared with the sequence of the *CYN* gene (GI: 6329170) that is available in the Nucleotide Database of the National Center for Biology Information (NCBI). The results obtained indicated that the right pTRA- K-CYN clones were generated and ready for *Agrobacterium tumefaciens* transformation.

### 3.2. Transformation of Agrobacterium Tumefaciens by pTRAK-CYN Vector

**Figure 4:**
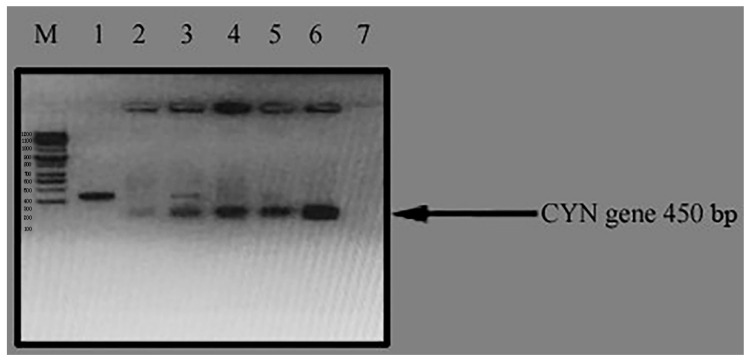
Colony PCR test for positive clones of *Agrobacterium tumefaciens* for pTRA-K-CYN. M: 100 bps DNA ladder; Lane 2, 3, 4, 5, 6: positive clones of *A. tumefaciens*; Lane 1, 7: negative clones of *A. tumefaciens*.

The plant expression vector, pTRA-K-CYN, was first transformed into competent *Agrobacterium tumefaciens* by the heat shock method. Then, the positive clones were determined using a PCR colony test (Figure 4).

### 3.3. Formation and Selection of Transgenic Chlamydomonas reinhardtii

**Figure 5:**
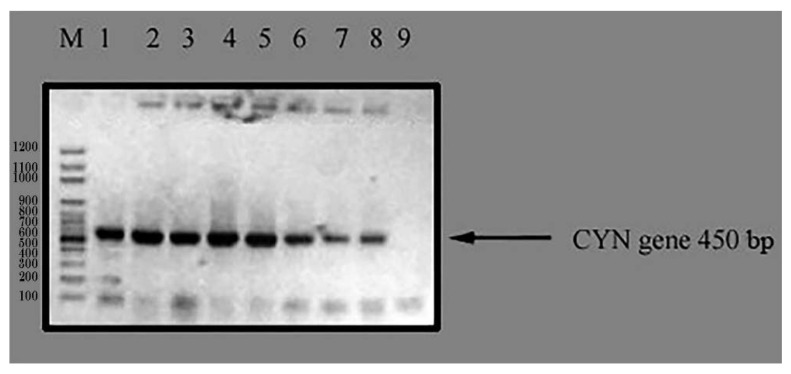
Colony PCR test of *CYN* transgenic algae. M: 100 bps DNA ladder; Lane 1: *CYN* positive control (transgenic *C. reinhardtii* contaiing *CYN* gene); Lane 2, 3, 4, 5, 6, 7, 8: transgenic *C. reinhardtii* algae tested with PCR system for *CYN* gene; and Lane 9: negative control (wild *C. reinhardtii)*.

**Figure 6:**
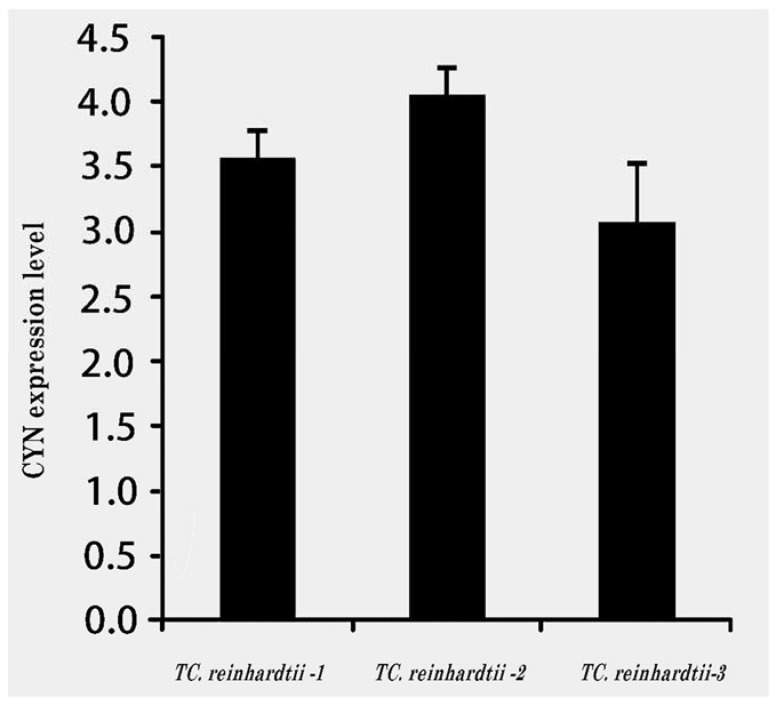
qRT-PCR analysis of *CYN* expression in three transgenic *C. reinhardtii* algae lines. Cycle threshold (Cts) value = 20.

Wild-type *C. reinhardtii* alga was transformed with the expression plasmid, pTRA-K-CYN, using a positive clone of *Agrobacterium tumefaciens* by the heat shock method. Two days after *C. reinhardtii* transformation, the cells were harvested. The selection of transgenic lines was performed on the DNA level using a colony PCR test for positive algae using specific primers for *CYN* transgenes against the negative control. The results of the colony PCR test declared the presence of seven different transgenic *C. reinhardtii* lines for the *CYN* gene (Figure 5). Three independent lines (2, 4, and 5; Figure 6) were randomly selected and named *TC. reinhardtii*-*1*, *TC. reinhardtii-2*, and *TC. reinhardtii-3.* They were used for quantitative real-time PCR analysis.

### 3.4. Quantitative Real-Time PCR Analysis for the Expression of CYN Genes in Three Transgenic C. reinhardtii

The total RNA was isolated from transgenic *C. reinhardtii***,** and was used for the synthesis of cDNA. The synthesized cDNA quality was tested by a PCR test using ACTIN2 RT-PCR primers. All the synthesized cDNA samples showed positive results after PCR analysis. These cDNA samples were used for the qRT-PCR analysis for *CYN* expression in transgenic-type *C. reinhardtii*.

The results of qRT-PCR analysis for the expression of *CYN* genes in three transgenic *C. reinhardtii* revealed the variable expression level for *CYN* genes observed among the three tested transgenic lines (*TC. Reinhardtii*-*1*, *TC. Reinhardtii-2*, and *TC. reinhardtii-3*). All the tested transgenic *C. reinhardtii* algae revealed a high expression level of the mRNA transcript accumulation of *CYN*, as compared with Actin2. The transgenic line, *TC. Reinhardtii-2*, showed the relatively highest level of *CYN*, followed by *TC. reinhardtii-1*, and then the *TC. reinhardtii-3* line (Figure 6). The transgenic line, *TC. Reinhardtii-2*, was used in further experiments with wild-type *C. reinhardtii*.

### 3.5. Comparison between Growth Parameters of Wild and Transgenic TC. reinhardtii-2 Lines under Potassium Cyanide Stress

**Figure 7:**
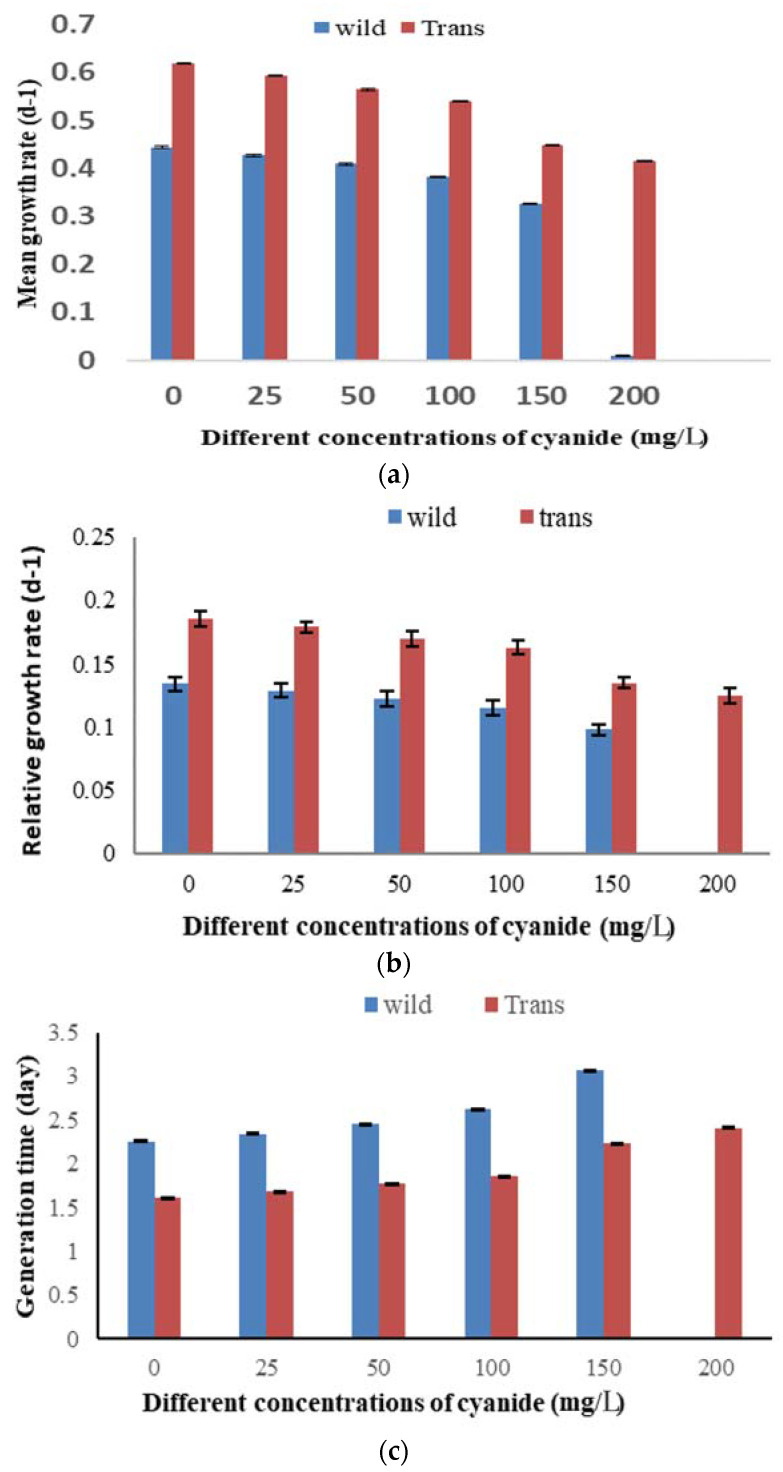
Growth parameters of wild-type and transformed *Chlamydomonas reinhardtii* under the effect of different KCN concentrations. (**a**) Mean growth rate, (**b**) relative growth rate, (**c**) generation time. Bars are mean ± standard error of three replicates. *p* < 0.001.

The growth parameter results revealed that there was an inverse relationship between the increase in potassium cyanide concentration and the mean growth rate, relative growth rate, and generation time of both wild and transgenic lines, but the transgenic line showed a better resistance and growth frequency in the presence of different potassium cyanide concentrations, as compared to the wild type (Figure 7a–c).

### 3.6. Mean Growth Rate

There was a gradual decrease in the growth rate because of the inhibitory effect of potassium cyanide. The maximum inhibition of the growth rate (R = 0.326 and 0.415 d^−1^) was recorded by treating the wild type with 150 mg/L KCN and transgenic cells with 200 mg/L KCN, as compared to the corresponding control (R = 0.444 and 0.619 d^−1^), respectively. A sudden growth depression in wild-type cells was notable with 200 mg/L KCN (Figure 7a).

### 3.7. Relative Growth Rate

The maximum inhibition of the relative growth rate (K′ = 0.098 and 0.125 d^−1^) was recorded by treating the wild type with 150 mg/L KCN and transgenic cells with 200 mg/L KCN, as compared to the corresponding control (K′ = 0.0.134 and 0.186 d^−1^) (Figure 7b).

### 3.8. Generation Time

The generation time of wild and transgenic *TC. reinhardtii-2* cells was increased under KCN. The maximum value of generation time was recorded by the highest KCN concentration of 150 mg/L for the wild type and 200 mg/L for the transgenic type (3.1 days and 2.4 days), as compared to the corresponding control (2.3 days and 1.6 days) (Figure 7c).

### 3.9. Bioremediation of Potassium Cyanide by Wild and Transgenic Line (TC. reinhardtii-2)

Transgenic *TC. reinhardtii-2* was significantly (*p* ≤ 0.001) able to bioremediate KCN from the given cyanide polluted samples, as compared with the wild type. In the presence of 25 mg/L and 50 mg/L, the removal ratio of KCN reached 100% by *TC. reinhardtii-2* after 36 h and 48 h respectively, whereas the wild type reached 100% removal after 48 h in presence of 25 mg/L, and reached only 21.43% after 12 h in presence of 50 mg/L. In the presence of 100 mg/L, 150 mg/L, and 200 mg/L, the removal ratio of KCN by *TC. reinhardtii-2* was gradually decreased, but *TC. Reinhardtii-2* still had the ability to grow. In contrast, the wild type was able to remove KCN with a very low ratio in the presence of 100 mg/L and 150 mg/L, and was not able to grow in the presence of 200 mg/L (Table 1).

## 4. Discussion

The enhancement of an ecofriendly method to remediate the pollutants from water and soil has become the main target of several recent studies, to avoid the chemical hazards released during the chemical treatment of pollutants. As several plants, bacteria, and fungi may utilize cyanide pollutants as a source of nitrogen for their metabolic processes, the bioremediation of cyanide investigations is becoming more significant than chemical treatment methods [32]. The high and significant efficiency of the algal bioremediation of cyanide has been proved previously, due to their volume ratios, efficient uptake, and storage systems [12]. The present study aimed to produce a transgenic form of *Chlamydomonas reinhardtii* by the transformation method via *Agrobacterium tumefaciens* for the cyanobacterial cyanase gene (CYN gi16329170) to remediate cyanide pollutants from polluted water. The vector, pTRAK-CYN, which carried the coding gene for cyanobacterial *CYN*, was transferred into *Agrobacterium tumefaciens*, and then the positive *Agrobacterium tumefaciens* was used to transform *C. reinhardtii* via *Agrobacterium*-*tumefaciens*-mediated transformation [19]. The cyanase enzyme can remediate cyanide and convert cyanide into carbon dioxide and ammonia [15]. The cyanase coding gene (*CYN*) was previously identified and genetically expressed in *Arabidopsis thaliana* [33,34]. The *CYN* gene was previously identified among bacteria, fungi, and plants. The transcriptional regulation and enzymatic activity of the *CYN* gene were primarily examined in *Escherichia coli* strain, B/1 [35]. The *CYN* gene was particularly prevalent among cyanobacteria, including numerous *Prochlorococcus* and *Synechococcus* strains [36].

In the present study, the *CYN* gene mRNA transcripts were quantified in three transgenic *C. reinhardtii* lines by qRT-PCR. All transgenic lines gave a high expression of *CYN* gene mRNA transcripts, as compared with the wild type. *TC. reinhardtii-2* showed the best result and gave the highest expression level. It was previously reported that a high expression of transgenes in *C. reinhardtii* could be increased when using genes with high GC content [37]. The GC content of the cyanobacterial *CYN* gene is 47%; this high GC content could be explained by the observed high expression levels of the *CYN* gene mRNA transcripts in the transgenic lines, as compared with the wild type. The gene expression in *C. reinhardtii* can be controlled by different promoters. Cauliflower mosaic virus 35S promoter (CaMV 35S) showed an induction of high transgene expression in *C. reinhardtii* [38]. Moreover, Cerutti et al. [39] declared that the RbcS2 (the small subunit of the ribulose bisphosphate carboxylase) promoter is used for the induction of transgene expression in *C. reinhardtii*, whereas Rosales-Mendoza et al. [20] reported other constitutive promoters, such as TubA1, AtpC, β-tub, PsaD, and Hsp70A, which induce high transgene expression in vectors used for the transformation of *C. reinhardtii*.

The expression efficiency of the cyanase enzyme in transgenic lines, compared with the wild type, was determined in our previously published work [21]. The results declared that cyanase activity was higher in the transgenic line than the wild type. The maximum cyanase activity in transgenic lines reached eight folds more than the wild type. Elgammal et al. [21] reported that cyanase activity may depend on the action of endogenous cyanase and other nitrogen assimilating enzymes; thus, it can be concluded that the transgenic *TC. reinhardtii-2* produces a high expression level of cyanase because of the presence of the *cyanase* gene coding sequence from *Synechococcus elongatus* (PCC 6803).

To assess the efficiency of the transgene CYN under cyanate toxicity, three growth parameters (mean growth rate, relative growth rate, and generation time) were evaluated using *TC. reinhardtii-2* and the wild type. The noticeable decline in growth parameters of the wild type in the presence of different KCN concentrations could possibly be due to the presence of an inadequate amount of the endogenous cyanase to remediate the external concentrations of KCN in the medium [33]. On the contrary, the presence of the cyanobacterial cyanase transgene in *TC. reinhardtii-2* improves the ability of *TC. reinhardtii-2* to produce a high expression level of cyanase to remediate high external concentrations of KCN in the medium, converting it to usable compounds for algal growth [21]. Similar growth performances were proved for the *CYN* transgenic and wild type of the *Arabidops* is *thaliana* plant [34]. The expression of the cyanobacterial cyanase in *A. thaliana* improves the plant’s ability to degrade the exogenous concentrations of cyanate on foliar parts of the plant, and improves root hair formation.

The transgenic algae under investigation can tolerate different ranges of cyanide, especially compared to the wild type. This may be due to the overexpression of cyanase in the *TC. reinhardtii-2* line, which can decay cyanide into NH_3_ and CO_2_ [40]. From the present results, the contact time is an important factor for KCN removal. There was a direct relationship between the removal degradation and the contact time and KCN concentration in the case of *TC. reinhardtii-2*, especially at low concentrations (25 and 50 mg/L). *TC. reinhardtii-2* can bioremediate KCN from exogenously applied cyanide, demonstrating the functioning of the cyanobacterial cyanase enzyme in *TC. reinhardtii-2*, whereas in the case of the wild type, it can bioremediate KCN at a lesser frequency; this could be due to the inadequate amount of the endogenous cyanase [33]. Moreover, the high KCN concentration (200 mg/L) caused an inverse relationship in *TC. reinhardtii-2*, and the wild type died. Gurbuz et al. [12] pointed out that the detoxification of KCN by algal cells was significantly influenced by the initial concentration of KCN. This may be because the accumulation of KCN by algal cells would achieve saturation in certain exposure concentrations [17]. High concentrations of cyanide and related compounds could pose a hazard to membrane integrity once the cell is destroyed [41]. In this regard, the wild type was damaged and consequently died upon exposure to the higher cyanide concentration (200 mg/L). As a result, *TC. reinhardtii-2* showed the ability to grow and to remediate KCN even in a low frequency following the exposure to a high concentration of cyanide. This conclusion proves the significance of utilizing *CYN* transgenic *C. reinhardtii* in KCN bioremediation.

## 5. Conclusions

The present results provide an effective ecofriendly transgenic *Chlamydomonas* microalga for the cyanide bioremediation from polluted fresh water. Additionally, the growth parameters of cyanate-stressed transgenic *C. reinhardtii* showed that the presence of the cyanobacterial cyanase transgene in *TC. reinhardtii-2* enhanced the ability of *TC. reinhardtii-2* to produce high expression levels of cyanase to remediate high external concentrations of KCN in medium, converting it to usable compounds for algal growth.

## Figures and Tables

**Table 1:** **Percentage of cyanide removal by wild-type and *TC. reinhardtii—2* lines**.

	Time	Type of Alga	0 h	6 h	12 h	24 h	36 h	48 h	72 h
Conc. of KCN (mg/L)									
**25**		**Wild type**	00.00 ± 0.00	13.53 ± 0.33	24.93 ± 0.80	41.31 ± 0.36	20.39 ± 0.39	100 ± 0.00	100 ± 0.00
		***TC. reinhardtii-2***	00.00 ± 0.00	17.54 ± 0.13	29.18 ± 0.34	53.22 ± 0.23	100 ± 0.00	100 ± 0.00	100 ± 0.00
**50**		**Wild type**	00.00 ± 0.00	13.47 ± 0.32	21.43 ± 0.00	21.45 ± 0.00	16.56 ± 0.31	4.87 ± 0.39	1.18 ± 0.38
		***TC. reinhardtii-2***	00.00 ± 0.00	19.28 ± 0.57	32.01 ± 0.19	46.41 ± 1.05	20.60 ± 0.85	100 ± 0.00	10.00 ± 0.00
**100**		**Wild type**	00.00 ± 0.00	12.50 ± 0.00	10.57 ± 0.71	19.01 ± 0.98	14.29 ± 0.29	9.37 ± 0.04	2.50 ± 0.13
		***TC. reinhardtii-2***	00.00 ± 0.00	19.19 ± 0.69	13.12 ± 1.02	26.24 ± 1.00	10.03 ± 1.04	20.62 ± 0.16	8.33 ± 0.46
**150**		**Wild type**	00.00 ± 0.00	12.56 ± 0.53	11.82 ± 0.45	10.32 ± 0.07	8.90 ± 0.73	0.86 ± 0.72	0.59 ± 0.00
		***TC. reinhardtii-2***	00.00 ± 0.00	18.04 ± 1.00	22.15 ± 0.96	24.97 ± 0.28	13.10 ± 0.00	11.48 ± 0.95	2.16 ± 0.56
**200**		**Wild type**	00.00 ± 0.00	00.00 ± 0.00	00.00 ± 0.00	00.00 ± 0.00	00.00 ± 0.00	00.00 ± 0.00	00.00 ± 0.00
		***TC. reinhardtii-2***	00.00 ± 0.00	16.16 ± 0.00	13.80 ± 0.02	13.40 ± 0.25	11.36 ± 0.98	9.30 ± 0.37	1.38 ± 0.14

Data are the average of three replicates ± SE.

## Data Availability

Most data generated or analyzed during this study are included in this published article.
